# Methodological considerations on the estimation of non-initial radiation effects among Hiroshima atomic bomb survivors: a commentary on Ohtaki *et al.* (2025)

**DOI:** 10.1093/jrr/rrag052

**Published:** 2026-07-21

**Authors:** Hideto Takahashi, Koji Ono

**Affiliations:** Faculty of Medicine, University of Tsukuba, 1-1-1 Tennodai, Tsukuba, Ibaraki 305-8575, Japan; Comprehensive Education Center, Tokyo Healthcare University, 2-5-1 Higashigaoka, Meguro-ku, Tokyo 152-8558, Japan

## Abstract

A recent study by Ohtaki *et al.* (Frontiers in Public Health, 2025) proposed that non-initial (residual) radiation contributed substantially to solid cancer mortality among Hiroshima atomic bomb survivors, estimating residual radiation doses exceeding 2 Sv in areas west of the hypocenter. This commentary examines four methodological considerations relevant to interpreting these findings. First, the study infers residual radiation dose from the same excess relative risk (ERR) used to assess cancer risk—a form of circular reasoning that precludes independent validation of the dose–response relationship. Second, the attribution of geographically asymmetric cancer mortality to a specific exposure mechanism is difficult to substantiate without individual-level data on potential confounders; a simple sensitivity analysis suggests that modest non-zero ERR in the assumed control region could substantially reduce the reported excess. Third, the cohort entry criterion of confirmed Hiroshima residency in 1970, approximately 25 years post-bombing, may introduce survivor and migration bias. Fourth, sex-differential behavioral factors following the bombing may confound the interpretation of sex-specific ERR estimates. These considerations do not negate the importance of the research question but suggest that the magnitude of residual radiation effects proposed by Ohtaki *et al.* warrants careful evaluation. Independent dosimetry and individual-level data are needed to clarify the role of residual radiation in long-term health outcomes among atomic bomb survivors.

## INTRODUCTION

The Life Span Study (LSS) of atomic bomb survivors, conducted by the Radiation Effects Research Foundation (RERF), has provided the primary epidemiological basis for international radiation risk assessment and radiation protection standards [1–3]. A recent article by Ohtaki *et al.* [4] investigated the potential contribution of non-initial (residual) radiation exposure to solid cancer mortality among Hiroshima survivors using data from the A-bomb Survivors Cohort Database (ABS) maintained at Hiroshima University. The authors reported notably higher excess relative risks (ERR) of solid cancer mortality in areas west of the hypocenter and inferred residual radiation doses exceeding 2 Sv for men in these western areas, representing a difference of two orders of magnitude compared with previously established dosimetry estimates of approximately 20–30 mGy (approximately equivalent to 20–30 mSv for γ-ray exposure) [5–7].

The geographic asymmetry of cancer mortality around the Hiroshima hypocenter is a well-recognized but incompletely explained phenomenon [8] and the question of residual radiation effects among atomic bomb survivors is scientifically important. The present commentary builds on previous work in this field [9], which reported significantly higher solid cancer mortality risks among early entrants to Hiroshima on the day of the bombing compared with those who entered three or more days later, using date of entry as a surrogate variable for residual radiation dose. The present commentary discusses four methodological considerations that are relevant to interpreting the findings of Ohtaki *et al.* [4].

## MATERIALS AND METHODS

This commentary is based on a critical appraisal of the study design, statistical methodology, and inferential framework reported by Ohtaki *et al.* [4]. The original study analyzed data from 43 056 atomic bomb survivors (17 603 men and 25 453 women) registered in the ABS database who were confirmed Hiroshima Prefecture residents as of 1 January 1970, were aged under 50 years at the time of the bombing and had been exposed within 5.0 km of the hypocenter. Solid cancer mortality was followed from 1970 to 2010. The authors estimated ERRs using multivariate survival analysis with an additive parametric hazard model under the linear no-threshold (LNT) hypothesis and inferred residual radiation doses by dividing the ERRs attributable to geographic location by the ERR per unit dose derived from initial radiation exposure.

For the present commentary, we examined: (i) the methodological validity of inferring radiation dose from the outcome variable; (ii) the attribution of spatially asymmetric cancer mortality to a specific exposure mechanism in the absence of individual-level confounders; (iii) potential sources of selection bias arising from the 1970 cohort entry criterion; and (iv) the interpretation of sex-specific ERR differences in the context of behavioral confounding. Published dosimetry reports [5–7] were used for comparison of residual dose estimates. For point (ii), a simple numerical sensitivity analysis was performed using the parameter estimates reported in Table 5 of Ohtaki *et al.* [4].

## RESULTS AND DISCUSSION

### Circularity in residual dose estimation

A central methodological concern relates to the estimation of residual radiation dose. Ohtaki *et al.* [4] inferred residual doses by dividing the ERR attributed to geographic location by the ERR per unit dose from initial radiation exposure. In conventional radiation epidemiology, radiation dose is an independent exposure variable used to estimate health risk as the outcome. In the approach adopted by Ohtaki *et al.*, however, exposure magnitude is derived from the outcome itself (observed excess cancer mortality) and the derived quantity is subsequently used to characterize the nature of the exposure. In epidemiological terms, this is analogous to estimating exposure from outcome data and then using the estimated exposure to explain the same outcome—a form of circular reasoning that precludes independent validation of the dose–response relationship.

Furthermore, the inferred residual doses exceeding 2 Sv are orders of magnitude larger than values estimated by established physical dosimetry systems [5–7]. While the authors attribute this discrepancy to inhalation of radioactive microparticles not accounted for in previous estimates, this hypothesis requires independent physical or biological validation before the inferred dose magnitudes can be treated as established exposure estimates.

### Spatial epidemiological inference and confounding

The study interprets higher cancer mortality west of the hypocenter as evidence of residual radiation transported by easterly wind and deposited through rainfall. However, spatial variation in disease outcomes may arise from multiple factors beyond environmental radiation exposure, including population density, socioeconomic conditions, residential migration patterns, occupational exposures, and health-related behaviors. Without individual-level information on these factors, attributing spatial differences in risk to a specific exposure mechanism remains ecologically inferential. It is worth noting that the appropriateness of control region selection has been a longstanding methodological concern in atomic bomb survivor epidemiology [8].

A critical assumption underlying the analysis is that the control region (RE, located east-southeast of the hypocenter beyond 2.5 km) was effectively shielded from residual radiation by the Hijiyama hill. This assumption is central to the study design, yet it is not independently verified. To illustrate the potential impact of this assumption, we conducted a simple sensitivity analysis using the parameter estimates from Table 5 of Ohtaki *et al.* [4]. If the RE control region had a non-zero residual ERR of 0.10–0.20 (comparable to the eastern semi-annular areas visible in Fig. 3C of the original article), the apparent excess ERR in the western RD region for men would be reduced by approximately 19–38% and the estimated residual dose would decrease from the reported 4.3 Sv to approximately 2.7–3.5 Sv. More notably, under the assumption of a residual ERR of 0.15 or greater in the control region, the excess ERR for women in the western RC region (reported as statistically significant at *P* < 0.05) would become statistically non-significant. These calculations suggest that the magnitude and statistical significance of the reported findings are sensitive to the validity of the control region assumption.

### Survivor bias and cohort entry at 25 years post-bombing

The cohort was defined as individuals confirmed to be Hiroshima Prefecture residents in 1970, approximately 25 years after the atomic bombing. This entry criterion may introduce survivor bias if individuals who received higher radiation doses, whether initial or residual, were less likely to survive to 1970, or if post-war residential patterns were associated with health status. Differential migration away from the most affected areas in the western districts could also distort the geographic distribution of survivors available for follow-up, potentially attenuating or inflating the observed spatial risk patterns in a non-random manner. These potential biases are not formally addressed in the original analysis.

### Sex differences and behavioral confounding

The study reports notably higher estimated residual radiation doses for men than for women across most geographic areas, which the authors attribute to greater male engagement in rescue and search activities immediately after the bombing. While this behavioral explanation is plausible, behavioral differences between men and women following the bombing could also influence long-term health outcomes through pathways other than radiation exposure, including nutritional status, psychological stress, occupational exposures during reconstruction, and smoking history. These factors were not explicitly addressed as potential confounders of the sex-specific ERR estimates, which complicates the attribution of sex differences in ERR solely to differential residual radiation exposure.

## CONCLUSION

The study by Ohtaki *et al.* [4] raises a scientifically important hypothesis regarding the potential contribution of non-initial radiation to solid cancer mortality among Hiroshima atomic bomb survivors and the geographic asymmetry of cancer mortality around the hypocenter merits continued investigation. However, four methodological considerations—circularity in dose estimation, spatial ecological inference in the absence of individual-level confounders, potential survivor and migration bias from the 1970 cohort entry criterion and behavioral confounding of sex-specific ERR estimates—limit the strength of inference that can be drawn from the current analysis. In particular, our sensitivity analysis demonstrates that the key assumption regarding the control region’s shielding from residual radiation has a material impact on both the magnitude and statistical significance of the reported findings.

Because studies of atomic bomb survivors have served as a foundational basis for international radiation risk assessment and radiation protection standards, careful methodological scrutiny of newly proposed hypotheses is essential. Future studies that incorporate independent physical or biological dosimetry for residual radiation, individual-level behavioral and socioeconomic data and analytical approaches that are robust to survivor selection effects would substantially strengthen the evidence base for evaluating the long-term health consequences of residual radiation exposure.

## PRESENTATION AT A CONFERENCE

None declared.
